# Measuring Glutathione Redox Potential of HIV-1-infected Macrophages*[Fn FN2]

**DOI:** 10.1074/jbc.M114.588913

**Published:** 2014-11-18

**Authors:** Ashima Bhaskar, MohamedHusen Munshi, Sohrab Zafar Khan, Sadaf Fatima, Rahul Arya, Shahid Jameel, Amit Singh

**Affiliations:** From the ‡ Department of Microbiology and Cell Biology, Centre for Infectious Disease and Research, Indian Institute of Sciences, Bangalore 560012,; the ¶ International Centre for Genetic Engineering and Biotechnology, New Delhi 110 67, and; the § Department of Biotechnology, Jamia Millia Islamia, New Delhi 25, India

**Keywords:** AIDS, Human Immunodeficiency Virus (HIV), Mycobacterium tuberculosis, Pathogenesis, Redox Signaling

## Abstract

Redox signaling plays a crucial role in the pathogenesis of human immunodeficiency virus type-1 (HIV-1). The majority of HIV redox research relies on measuring redox stress using invasive technologies, which are unreliable and do not provide information about the contributions of subcellular compartments. A major technological leap emerges from the development of genetically encoded redox-sensitive green fluorescent proteins (roGFPs), which provide sensitive and compartment-specific insights into redox homeostasis. Here, we exploited a roGFP-based specific bioprobe of glutathione redox potential (*E*_GSH_; Grx1-roGFP2) and measured subcellular changes in *E*_GSH_ during various phases of HIV-1 infection using U1 monocytic cells (latently infected U937 cells with HIV-1). We show that although U937 and U1 cells demonstrate significantly reduced cytosolic and mitochondrial *E*_GSH_ (approximately −310 mV), active viral replication induces substantial oxidative stress (*E*_GSH_ more than −240 mV). Furthermore, exposure to a physiologically relevant oxidant, hydrogen peroxide (H_2_O_2_), induces significant deviations in subcellular *E*_GSH_ between U937 and U1, which distinctly modulates susceptibility to apoptosis. Using Grx1-roGFP2, we demonstrate that a marginal increase of about ∼25 mV in *E*_GSH_ is sufficient to switch HIV-1 from latency to reactivation, raising the possibility of purging HIV-1 by redox modulators without triggering detrimental changes in cellular physiology. Importantly, we show that bioactive lipids synthesized by clinical drug-resistant isolates of *Mycobacterium tuberculosis* reactivate HIV-1 through modulation of intracellular *E*_GSH_. Finally, the expression analysis of U1 and patient peripheral blood mononuclear cells demonstrated a major recalibration of cellular redox homeostatic pathways during persistence and active replication of HIV.

## Teaser

**Background:** Abnormal glutathione poise has been associated with HIV-1 infection; however, the underlying importance is unclear.

**Results:** Measurement of glutathione-redox potential (*E*_GSH_) revealed higher capacity of latently infected cells to resist oxidative stress and apoptosis, whereas HIV-1 replication perturbed glutathione homeostasis.

**Conclusion:** Dynamic changes in *E*_GSH_ regulate HIV-1 persistence and reactivation.

**Significance:** Glutathione-redox signaling plays a critical role in HIV-1 infection.

## Introduction

An estimated 25 million people have died due to AIDS and ∼36 million people are currently infected with its causative agent, human immunodeficiency virus type-1 (HIV-1). Because HIV-1 can persist in a latent state for many years and then reactivates to cause immunodeficiency, our particular interest is to understand the mechanisms underlying reactivation from latency. In this context, redox signaling is thought to be one of the main mechanisms regulating HIV-1 replication, immune dysfunction, and disease development (1). Interestingly, one of the foremost consequences of increased oxidative stress is the reactivation of HIV from latent reservoirs via NF-κB-mediated transcriptional activation of the viral long terminal repeat (LTR) (2, 3). Oxidative stress also modulates immunopathogenesis of HIV-1 infection by disturbing adaptive immune mechanisms, including decreased immune cell proliferation, loss of memory T-cell response, and premature T_reg_ response (4–6).

A major cellular antioxidant, glutathione (GSH), provides an abundant source of reducing equivalent and functions as a major protective system against oxidative stress (7). Therefore, the majority of studies linking redox stress with HIV-1 infection demonstrates variations in GSH levels in infected cells and tissues (8, 9). Additionally, a few studies have been performed wherein the levels of reactive oxygen species (ROS)^3^ and reactive nitrogen species (RNS) were detected as the signatures of redox stress during HIV-1 infection (10–13). However, all glutathione redox research has been based on chemical analyses of whole-cell or tissue extracts (14), which inevitably destroy subcellular compartment-specific information and do not provide any real time assessment of redox changes associated with the progression of infection. Alternatively, measurements of ROS and reactive nitrogen species are entirely based on harnessing the potential of oxidant-sensitive cell-permeable dyes such as 2′,7′-dichlorodihydrofluorescein diacetate, which suffer from inherent nonspecificity and can get oxidized even in the absence of ROS by metals, peroxidases, and cytochrome *c* (15–17). Because of these limitations, real time determination of redox potential of HIV-1-infected cells has never been performed. This is a critical knowledge gap that has adversely affected our ability to comprehensively study the redox mechanisms of HIV-1 infection and has hindered the development of novel redox-based intervention strategies against HIV/AIDS. Therefore, the application of technologies to precisely measure temporal and compartment-specific resolution of dynamic changes in intracellular redox potential of HIV-1-infected cells has the potential to overcome many of the deficiencies in our understanding of the redox basis of HIV infection and may enable high throughput screens to identify small molecule modulators of intracellular redox homeostasis to control HIV-1 infection.

In this work, we describe the application of a genetically encoded glutathione biosensor comprising human glutaredoxin-1 linked to a redox-sensitive green fluorescent protein (Grx1-roGFP2) in accurately measuring glutathione redox potential (*E*_GSH_) (18) of cytosol and mitochondria of the HIV-1-infected monocytic cell line (U1) during different phases of infection. Based on these findings, the contribution of *E*_GSH_ in regulating resistance of U1 toward apoptosis is examined. Importantly, this technology also allowed us to address the redox basis of synergy between HIV-1 and *Mycobacterium tuberculosis*. Finally, our data provide a highly resolved view of how fluctuations in *E*_GSH_ modulate expression of antioxidant genes and redox pathways during latent and active phases of infection. The physiological significance of these findings was validated by examining the expression of redox genes in the peripheral blood mononuclear cells (PBMCs) of HIV-1-infected patients. Our study illustrates the utility of genetically encoded redox biosensors in dissecting redox mechanisms associated with HIV-1 infection that will lead to the development of better strategies for the control of HIV-1 replication, progression, and disease development.

## EXPERIMENTAL PROCEDURES

### 

#### 

##### Generation of Biosensor Expression Constructs

The Grx1-roGFP2-containing vector was obtained from Tobias P. Dick (19). The Grx1-roGFP2 open reading frame (ORF) was PCR-amplified and cloned in pMSCVpuro using BglII and HpaI to generate pMSCVpuro-Grx1-roGFP2. To target Grx1-roGFP2 to the mitochondria of the cell, Grx1-roGFP2 was cloned in fusion with the cytochrome *c* oxidase subunit VIIIA (Cox8A) leader sequence in pMSCVpuro-Grx1-roGFP2. Mitochondrial signaling peptide of Cox8A was amplified using the following primers: Cox8A_F 5′-TAAGATCTCGAGATGTCCGTCCTGACGCCGCTG-3′ and Cox8A_R 5′-TA**AGATCT**CAACGAATGGATCTTGGCGCGCGG-3′. The bold letters represent the BglII site, and the underlined sequence represents the XhoI site. The amplified fragment was purified and cloned into the BglII site upstream of Grx1-roGFP2 in the pMSCVpuro vector to generate pMSCVpuro-mito-Grx1-roGFP2. Restriction digestion and DNA sequencing verified the construction of recombinant vectors. These vectors along with the helper plasmids (pVSVg and pGag-Pol) were used to prepare virus stocks for transduction experiments.

##### Stable Cell Line Generation and Flow Cytometry

Various cell lines stably expressing the Grx1-roGFP2 biosensors were generated by lentiviral transduction and subsequent selection with 350 ng/ml puromycin (20). The ratiometric response of cells expressing the Grx1-roGFP2 sensor was obtained by measuring excitation at 405 and 488 nm at a fixed emission (510/10 nm) on a FACS Verse Flow cytometer (BD Biosciences). Data were analyzed using the FACSuite software. For analyzing *M. tuberculosis-*infected macrophages, cells were washed with 1× phosphate-buffered saline (PBS) and treated with 10 mm *N*-ethylmaleimide (NEM) for 5 min (to block the thiol state of roGFP2) (21) followed by fixation with 4% paraformaldehyde (PFA) for 15 min and flow cytometry.

##### Mammalian Cells, Bacterial Cultures, and Infection

We grew human embryonic kidney 293T cells (ATCC, Manassas, VA), the human monocytic U937 and T-lymphocytic Jurkat cells (ATCC, Manassas, VA), and the chronically infected U1 and J1.1 cells (AIDS Research and Reference Reagent Program, National Institutes of Health) in culture medium as recommended by the ATCC. For differentiation and HIV-1 activation, U937 and U1 cells were treated with 5 ng/ml phorbol 12-myristate 13-acetate (PMA; Sigma) for 12 h. The bacterial strains used in this study were as follows: *M. tuberculosis* H37Rv and the field isolates Jal 2287 and MYC 431 (kind gift from Dr. Kanury V.S. Rao, ICGEB, New Delhi, India). Bacteria were grown in Middlebrook 7H9 broth (Difco) supplemented with 10% (v/v) oleic acid albumin dextrose catalase (BD Biosciences), 0.1% (v/v) glycerol, and 0.1% (v/v) Tween 80 until the mid-log phase (*A*_600__nm_ of 0.8). The PMA-differentiated U937 cells were seeded at 2 × 10^5^ cells per well in 24-well plates and infected with *M. tuberculosis* strains H37Rv, Jal 2287, and MYC 431 at a multiplicity of infection (m.o.i.) of 10 for 4 h. Extracellular bacteria were removed by washing twice with 1× PBS.

##### Redox Potential Measurements

The intracellular redox potential measurements were done as described earlier (18). For each experiment, the minimal and maximal fluorescence ratios were determined, which correspond to 100% sensor reduction and 100% sensor oxidation, using DTT (10 mm) as the reductant and H_2_O_2_ (10 mm) as the oxidant, respectively. The observed fluorescence ratio was then used to calculate the corresponding degree of sensor oxidation using Equation 1.

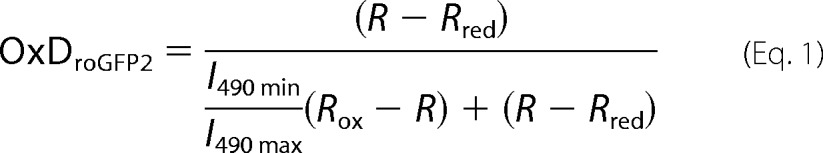
 Where *R* is the observed ratio; *R*_red_ and *R*_ox_ are the ratios of completely reduced and oxidized roGFP2, respectively; *I*_490 min_ and *I*_490 max_ are the fluorescence intensities measured with excitation at 490 nm for fully oxidized and fully reduced roGFP2, respectively. Next, the intracellular sensor redox potential *E*_roGFP2_ was calculated, using Nernst Equation 2,

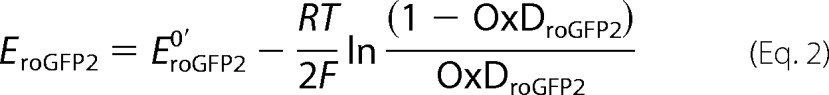
 where roGFP2 has an average consensus midpoint redox potential of *E*_roGFP2_^0′^ = −280 mV (19). Based on the equilibration between the sensor and the glutathione redox couple, we obtain the glutathione redox potential *E*_GSH_ = *E*_roGFP2_.

##### HIV-1 p24 Staining

For intracellular p24 staining, cells were washed once with FACS staining buffer (1× PBS containing 3% fetal bovine FBS) followed by fixation and permeabilization using the fixation/permeabilization kit (eBiosciences). After washing twice with FACS-staining buffer, permeabilized cells were incubated with 100 μl of a 1:100 dilution of phycoerythrin-conjugated mouse anti-p24 mAb (KC57-RD1; Beckman Coulter, Inc.) for 30 min at 4 °C. After two additional washes, cells were analyzed with BD FACS Verse Flow cytometer (BD Biosciences), and data analysis was performed with FACSuite software. HIV production was also monitored in the culture supernatants with p24 ELISA (ABL, Inc.) according to the manufacturer's specifications.

##### Propidium Iodide Staining

H_2_O_2_-treated cells were washed, suspended in 1× PBS, and stained with 1.5 μm propidium iodide (PI) for 15 min in the dark. After washing twice with 1× PBS, cells were analyzed on a flow cytometer using the phycoerythrin detector with 488 nm excitation and 575/26 nm emission.

##### Detection of ROS

ROS were detected using the probe 5- (and 6)-chloromethyl-2′,7′-dichlorodihydrofluorescein diacetate (CMH_2_DCF-DA; Invitrogen), which is a nonfluorescent cell-permeable compound. Within the cell, esterases cleave the acetate groups on CMH_2_DCF-DA, thus trapping the reduced probe 2′,7′-dichlorodihydrofluorescein (DCFH) intracellularly. ROS in the cells oxidize DCFH, yielding the fluorescent product 2′,7′-dichlorofluorescein (DCF). The fluorescent signal detected (excitation, 485 nm; emission, 530 nm) is proportional to ROS levels. Cells were loaded with 10 μmol liter^−1^ of DCF-DA followed by exposure to the indicated concentrations of H_2_O_2_ for 30 min, and relative ROS units were determined by flow cytometry.

##### Assessment of Apoptosis

Cells were treated with H_2_O_2_ in the presence or absence of l-buthionine sulfoximine (BSO) for 24 h, washed twice with 1× PBS, and the frequency of apoptotic cells was analyzed with annexin-V/PI staining as per manufacturer's instructions (Cayman). Annexin-V^+^/PI^−^ cells representing apoptotic cells were determined by flow cytometry using FITC (488 nm excitation and 520 nm emission) and phycoerythrin (488 nm excitation and 578 nm emission) channels.

##### GSH/GSSG Assay

Whole-cell glutathione, reduced glutathione (GSH), and oxidized glutathione (GSSG) were measured by using the glutathione assay kit (Cayman) according to the manufacturer's instructions.

##### Confocal Microscopy

Grx1-roGFP2 expressing cells were fixed with 4% PFA. For staining mitochondria, prior to fixing, the cells were pretreated for 1 h with MitoTracker (100 nm; Invitrogen). The coverslips were washed thoroughly with PBS and mounted onto glass slides with mounting media (Antifade reagent, Invitrogen). The Grx1-roGFP2 fluorescence was analyzed at 488 nm excitation and 525 nm emission, and MitoTracker-stained cells were visualized at 540 nm excitation and 630 nm emission.

##### Purification of Nef and Tat Proteins

HIV-1 subtype B Nef and subtype C Tat proteins were purified as described previously (22, 23).

##### Quantitative Real Time-PCR (qRT-PCR)

Total cellular RNA was isolated by RNeasy mini kit (Qiagen), according to the manufacturer's recommendations. RNA (500 ng) was reverse-transcribed to cDNA (iScript^TM^ cDNA synthesis kit, Bio-Rad), subjected to real time PCR (iQ^TM^ SYBR® Green Supermix, Bio-Rad), and performed using the Bio-Rad C1000^TM^ real time PCR system. The expression level of each gene is normalized to human β-actin. The oligonucleotides used are described in Table 1.

**TABLE 1:** **Sequences of oligonucleotides used to perform qRT-PCR**

Actin	Forward	5′-ATGTGGCCGAGGACTTTGATT-3′
	Reverse	5′-AGTGGGGTGGCTTTTAGGATG-3′
p24	Forward	5′-ATAATCCACCTATCCCAGTAGGAGAAAT-3′
	Reverse	5′-TTGGTTCCTTGTCTTATGTCCAGAATGC-3′
CAT	Forward	5′-TTAATCCATTCGATCTCACC-3′
	Reverse	5′-GGCGGTGAGTGTCAGGATAG-3′
GPX1	Forward	5′-CAACCAGTTTGGGCATCAG-3′
	Reverse	5′-GTTCACCTCGCACTTCTCG-3′
GPX2	Forward	5′-CCAGCTCAACGAGCTGCAATG-3′
	Reverse	5′-CCCCCAGGACGGACATACTT-3′
GPX3	Forward	5′-GCCGGGGACAAGAGAAGT-3′
	Reverse	5′-AAAAGGGGTGCATTGCACTG-3′
GPX4	Forward	5′-GCCTTCCCGTGTAACCAGT-3′
	Reverse	5′-GCGAACTCTTTGATCTCTTCGT-3′
GSR	Forward	5′-CTTGCGTGAATGTTGGATGT-3′
	Reverse	5′-GACCTCTATTGTGGGCTTGG-3′
GSS	Forward	5′-GCCAAAGCCTGGGAGCTCTA-3′
	Reverse	5′-CAAACAGCCTTCGGTCTTGGT-3′
HO2	Forward	5′-ATGTCAGCGGAAGTGGAAAC-3′
	Reverse	5′-CTCTGAGAGGTCAGCCATTC-3′
PRDX2	Forward	5′-CCCCTGACTTCAAGGCCACA-3′
	Reverse	5′-GAAGTCCTCTGCACGGTTGCTGA-3′
SOD1	Forward	5′-GGTCCTCACTTTAATCCTCTAT-3′
	Reverse	5′-CATCTTTGTCAGCAGTCACATT-3′
SOD2	Forward	5′-TGACAAGTTTAAGGAGAAGC-3′
	Reverse	5′-GAATAAGGCCTGTTGTTCC-3′

##### Oxidative Stress and Antioxidant Pathway Gene Expression Array

Total RNA was extracted from U1 and U937 cells using the Qiagen RNeasy mini kit (Qiagen). From this, 500 ng of RNA was reverse-transcribed into cDNA with the RT^2^ first strand kit (SABiosciences, Frederick, MD). Synthesized cDNA was subjected to the human oxidative stress and antioxidant defense PCR plus array (PAHS-065YD, SABiosciences) using the Bio-Rad C1000 real time PCR machine. PCR array data were analyzed by using SABiosciences software. The data were normalized to the housekeeping gene encoding human β-actin. Genes were considered to be up-regulated or down-regulated if changes in expression levels were >2- or <2-fold, respectively.

##### Lipid Extraction

Extractable polyketide lipids from *M. tuberculosis* strains H37Rv, Jal 2261, and MYC 431 were isolated as described previously (24). Total lipids were dissolved in diethyl ether and coated onto cell culture plates at a concentration of 50 μg/ml prior to addition of U937 monocytes.

##### Expression Analysis Using Patient PBMCs

Briefly, PBMCs were collected from symptomatic HIV/AIDS patients (*n* = 8) who were not on anti-retroviral therapy, with a mean age of 33 years and mean CD4 counts of <200/μl. The PBMCs from age-matched healthy controls (*n* = 6, average age 29) were also collected. The PBMCs were isolated from whole blood via Ficoll density gradient method followed by red blood cell lysis as described elsewhere (25). Total cellular RNA isolation, cDNA synthesis, and qRT-PCR analysis were performed as described above. The oligonucleotides used are described in Table 1.

##### Ethics Statement

For expression analysis, RNA samples isolated from the PBMCs of symptomatic HIV/AIDS patients and healthy controls were utilized. Whole blood was collected from HIV/AIDS patients recruited from the National AIDS Control Organization Anti-retroviral Therapy Clinics at Dr. Ram Manoharan Lohia Hospital and Maulana Azad Medical College Hospital in New Delhi, India. Ethics committees at the participating institutions and the National AIDS Control Organization, New Delhi, India, approved the study. All subjects were HIV sero-positive; those on anti-tubercular therapy and/or anti-retroviral therapy were excluded from the study. Potential study subjects were selected based on their case records, and the study was explained to them by one of the investigators in the presence of a social worker. Written informed consent was obtained from each participant before obtaining the samples.

##### Statistical Analysis

All data were derived from at least three independent experiments. Statistical analyses were conducted using GraphPad Prism software, and values were presented as mean ± S.D. The statistical significance of the differences between experimental groups was determined by two-tailed, unpaired Student's *t* test unless specified. Differences with a *p* value of <0.05 were considered significant.

## RESULTS

### 

#### 

##### Grx1-roGFP2 Biosensor

The Grx1-roGFP2 biosensor allows noninvasive imaging of the redox state of the glutathione redox couple (GSH/GSSG) inside subcellular compartments (18). It has two fluorescence excitation maxima at ∼400 and ∼490 nm, and the relative excitation intensities depend upon the thiol-disulfide state of the two cysteines engineered on the surface of the roGFP2 β-barrel (19). The oxidation of cysteines generates a disulfide bond, which increases the fluorescence intensity at ∼400 nm along with a relative decrease at ∼490 nm, although an inverse response is detected upon reduction (19). Because the biosensor response is ratiometric, measurements are independent of changes in cell density and protein content. The specific equilibration of the roGFP2 dithiol-disulfide redox pair (roGFP2_red_/roGFP2_oxi_) with the glutathione redox pair (GSH/GSSG) is catalyzed by the covalently fused Grx1 moiety (19). Of note, the Grx1-roGFP2 bioprobe is restricted to measure *E*_GSH_ in a range of −240 to −320 mV (19). Thus, complete oxidation or reduction of the biosensor is not equivalent to full oxidation or reduction of the GSH pool inside the cell (19). Importantly, Grx1-roGFP2 responds to nanomolar levels of intracellular GSSG within a few minutes, making it a highly sensitive and rapid bioprobe for the dynamic imaging of *E*_GSH_.

##### Measuring Steady-state E_GSH_ of HIV-1-infected Monocytes

**FIGURE 1.**
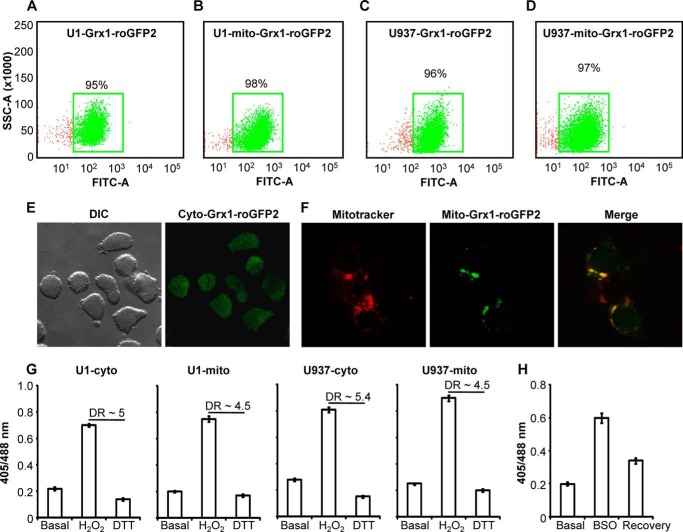
**Generation of transgenic cell lines expressing the biosensor.** Dot plot of U1 (*A* and *B*) and U937 cells (*C* and *D*) expressing the biosensor either in the cytosol or in the mitochondrial matrix. *E,* confocal image of U937 cells expressing Grx1-roGFP2 in the cytosol. *F,* mitochondrial targeting of the biosensor expressed in U937 cells. Grx1-roGFP2 is shown as *green*, and MitoTracker stain is *red,* and *yellow* signal demonstrates overlap. *G,* response of each cell line to exogenously applied oxidant H_2_O_2_ (10 mm) and reductant DTT (10 mm) for 5 min. *DR,* dynamic range. *H,* U937 cells expressing cytosolic *E*_GSH_ bioprobe were treated with 1 mm BSO for 24 h followed by recovery in fresh media for an additional 24 h. *Error bars* represent standard deviation from the mean (*n* = 3). *DIC*, differential interference contrast.

To image redox signaling during HIV-1 infection, we created stably transfected promonocytic U1 cells that express either a cytosolic (U1-Grx1-roGFP2) or a mitochondrial (U1-mito-Grx1-roGFP2) *E*_GSH_ bioprobe (Fig. 1, *A* and *B*). The U1 cell line is a well studied model of post-integration latency and was derived from a chronically infected clone of the parent promonocytic cell line U937 (26). It shows very low basal expression of two integrated copies of the HIV-1 genome, but gene expression and viral replication can be activated by various signals including PMA, tumor necrosis factor-α (TNF-α), interferon-γ (IFN-γ), granulocyte monocyte-colony-stimulating factor (GM-CSF), etc. (26, 27), making these cells a good model to study HIV-1 latency and reactivation (26). As an uninfected control, we stably expressed Grx1-roGFP2 in the cytosol and in the mitochondria of U937 cells (Fig. 1, *C* and *D*). Previous studies in various organisms have shown that cytosolic Grx1-roGFP2 and mito-Grx1-roGFP2 correctly localized to the cytosol and mitochondria, respectively (21). Using confocal microscopy, we also confirmed accurate subcellular targeting of the biosensor to either cytosol or mitochondria in our transgenic cells (Fig. 1, *E* and *F*). Next, using flow cytometry, we examined the ratiometric response (405/488 nm) of the biosensor toward exogenous addition of either the cell-permeable oxidant, hydrogen peroxide (H_2_O_2_), or the reductant, dithiothreitol (DTT). As shown in Fig. 1*G*, oxidation of Grx1-roGFP2 in cytosol and mitochondria of U1 and U937 cells by H_2_O_2_ exposure increased the 405/488 ratio, whereas DTT exposure had the opposite effect. The incubations with H_2_O_2_ and DTT indicate that the dynamic range of Grx1-roGFP2 in each case was 4.5–5.5, which is in agreement with studies done using Grx1-roGFP2 in the cytosol and mitochondria of other mammalian cells (18, 19). Notably, the 405/488 ratios of untreated and DTT-treated U1 and U937 transgenic cells were very close to each other, suggesting that Grx1-roGFP2 was nearly 100% reduced under steady-state conditions. Consistent with this, we found that *E*_GSH_ of cytosol and mitochondria in both U1 and U937 cells was highly reduced and in the range of −310 to −320 mV (see under “Experimental Procedures” for calculating *E*_GSH_) reaching the lower limit of the Grx1-roGFP2 biosensor of −320 mV (19). Finally, GSH specificity of the biosensor was confirmed by treating U937 cells expressing cytosolic Grx1-roGFP2 with 1 mm BSO, which lowers cellular GSH content by specifically inhibiting γ-glutamyl cysteine synthetase (γ-GCS) activity, thereby increasing the GSSG/GSH ratio and inducing an oxidative shift in *E*_GSH_ (28). The U937 cells pretreated with BSO showed a significant increase in the 405/488 ratio (Fig. 1*H*), indicating an oxidative shift in *E*_GSH_. Importantly, subsequent washout of BSO rapidly restored steady-state *E*_GSH_, thus confirming the specificity of the biosensor toward the GSH reductive pathway (Fig. 1*H*). Taken together, these results show that uninfected and chronically infected monocytic cells exhibit comparable steady-state *E*_GSH_ and suggest that the low basal levels of HIV-1 expression in U1 cells do not influence the cytosolic and mitochondrial redox state of these cells.

##### Monitoring Dynamic Changes in E_GSH_ of U1 Cells upon Oxidative Stress

**FIGURE 2.**
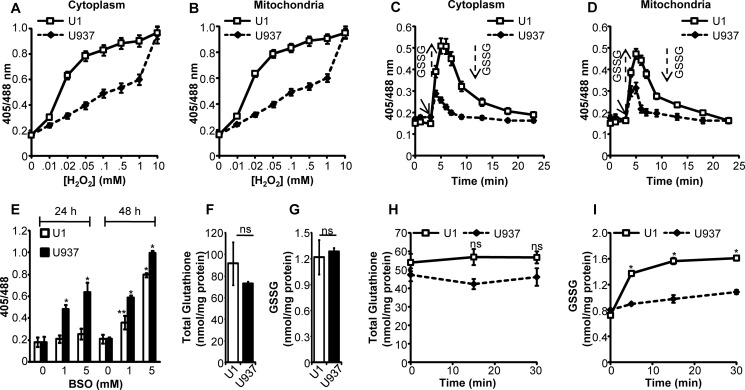
**Redox responsiveness of U1 and U937 cells toward H_2_O_2_.** U1 and U937 cells expressing cytosolic (*A*) and mitochondrial (*B*) *E*_GSH_ probes were treated with varying concentrations of H_2_O_2_ for 2 min, and the ratiometric sensor response was measured. U1 and U937 cells expressing cytosolic (*C*) and (*D*) mitochondrial biosensors were treated with 20 μm H_2_O_2_, and the ratiometric sensor response was measured over time. *Arrows* represent increase or decrease in subcellular GSSG pool in response to oxidative or anti-oxidative response, respectively. *E,* U1 and U937 cells expressing the cytosolic *E*_GSH_ bioprobe were treated with 1 and 5 mm BSO, and sensor response was measured at indicated time points. *p* values are calculated by comparing treated samples with untreated controls. Whole-cell total glutathione (*F*) and GSSG (*G*) concentrations were measured in lysates of unstressed U1 and U937 cells. U1 and U937 cells were treated with 20 μm H_2_O_2_, and whole-cell total glutathione (*H*) and GSSG (*I*) concentrations were measured at indicated time points. *Error bars* represent S.D. from the mean. All experiments are performed at least twice. *p* values were calculated by comparing U1 and U937 cells. *, *p* < 0.01; **, *p* < 0.05; *ns,* not significant.

The steady state *E*_GSH_ measurements do not reveal any information regarding the capacity of a cell to respond to oxidative challenge. Because HIV-1-infected individuals suffer from chronic oxidative stress (11), we monitored real time changes in subcellular *E*_GSH_ of U1 and U937 cells upon H_2_O_2_ exposure. The U1 and U937 cells were challenged with various concentrations of H_2_O_2_ for 2 min, and the sensor response was measured. We found that although treatment with ∼100 μm of H_2_O_2_ for 2 min was sufficient to attain nearly 90% oxidation of Grx1-roGFP2 in the cytosol and mitochondria of U1 cells, a similar response could only be observed with an ∼100-fold higher concentration of H_2_O_2_ (10 mm) in U937 cells (Fig. 2, *A* and *B*). Next, we measured Grx1-roGFP2 oxidation-reduction kinetics in response to a low concentration of H_2_O_2_ (20 μm). A fast and reversible change in the fluorescence ratio of Grx1-roGFP2 in the subcellular compartments of both U1 and U937 cells (Fig. 2, *C* and *D*) was detected. However, crucial differences between U1 and U937 were also apparent. For example, the cytosol and mitochondria of U937 cells showed only a fractional (405/488 ratio = ∼0.3) increase in the degree of Grx1-roGFP2 oxidation, whereas the apparent response in U1 subcompartments was much larger (405/488 ratio = ∼0.5) (Fig. 2, *C* and *D*). Consequently, the time required to recover from oxidative stress and achieve steady-state levels of Grx1-roGFP2 (405/488 ratio = ∼0.15) was faster in U937 as compared with U1 cells (Fig. 2, *C* and *D*). These results suggest a greater capacity of U1 to metabolize H_2_O_2_ by rapidly converting it into GSSG, a reaction likely mediated by endogenous glutathione peroxidases (19).

Inhibition of glutathione biosynthesis has been shown to perturb the redox poise of the GSH/GSSG couple to modulate biosensor response (29). To study whether U1 and U937 respond differently to GSH inhibition, we treated cells with 1 and 5 mm BSO for 24 and 48 h. No significant difference in the degree of Grx1-roGFP2 oxidation was observed in the BSO-treated U1 cells at 24 h post-treatment (Fig. 2*E*). In contrast, U937 showed a significant increase in the biosensor oxidation at 24 h post-treatment. Although at 48 h post-BSO treatment, both U937 and U1 showed a significantly oxidized *E*_GSH_, and Grx1-roGFP2 oxidation remained relatively high in U937 as compared with U1 cells (Fig. 2*E*). Together, these findings indicate that persistently infected monocytes are better equipped to sense and respond to redox imbalance induced by ROS and GSH depletion.

To compare the findings obtained from biosensor measurements with the widely used conventional technologies, we measured total glutathione content (GSH + GSSG) and GSSG concentration in U1 and U937 cells using chemical-enzymatic analysis of whole-cell extracts. Whole-cell glutathione content and GSSG concentration were not significantly different in U1 and U937 cells (*p* = 0.17 and 0.44, respectively), which agrees with our biosensor results demonstrating comparable steady-state *E*_GSH_ of these cells (Fig. 2, *F* and *G*). Furthermore, the GSH/GSSG ratio in U1 and U937 cells was found to be similar to the earlier reported GSH/GSSG ratio of 30–100:1 in unstressed mammalian cells (7). Next, we measured whole-cell glutathione and GSSG concentrations in U1 and U937 cells upon treatment with 20 μm H_2_O_2_. Whole-cell total glutathione concentration did not change upon H_2_O_2_ challenge in both the cell lines (Fig. 2*H*). In contrast, both U1 and U937 cells respond to oxidative stress by elevating total GSSG levels (Fig. 2*I*). Moreover, similar to the biosensor response, U1 cells showed a larger increase in GSSG concentration as compared with U937 cells (*p* < 0.01) (Fig. 2*I*). However, although Grx1-roGFP2 response showed a rapid recovery from oxidative stress (Fig. 2, *C* and *D*), whole-cell measurements did not detect any decrease in GSSG concentrations even after 30 min of exposure to H_2_O_2_ (Fig. 2*I*)_._ This discrepancy between biosensor response and total cellular GSSG analysis could be due to measurement of *E*_GSH_ in defined subcompartments (cytosolic and mitochondrial) in the former as compared with mixing of GSSG pools from various subcellular compartments in the latter. These findings highlight the advantage of using Grx1-roGFP2 over routinely used redox technologies for unambiguous determination of real time changes in *E*_GSH_ in specific subcellular compartments.

**FIGURE 3.**
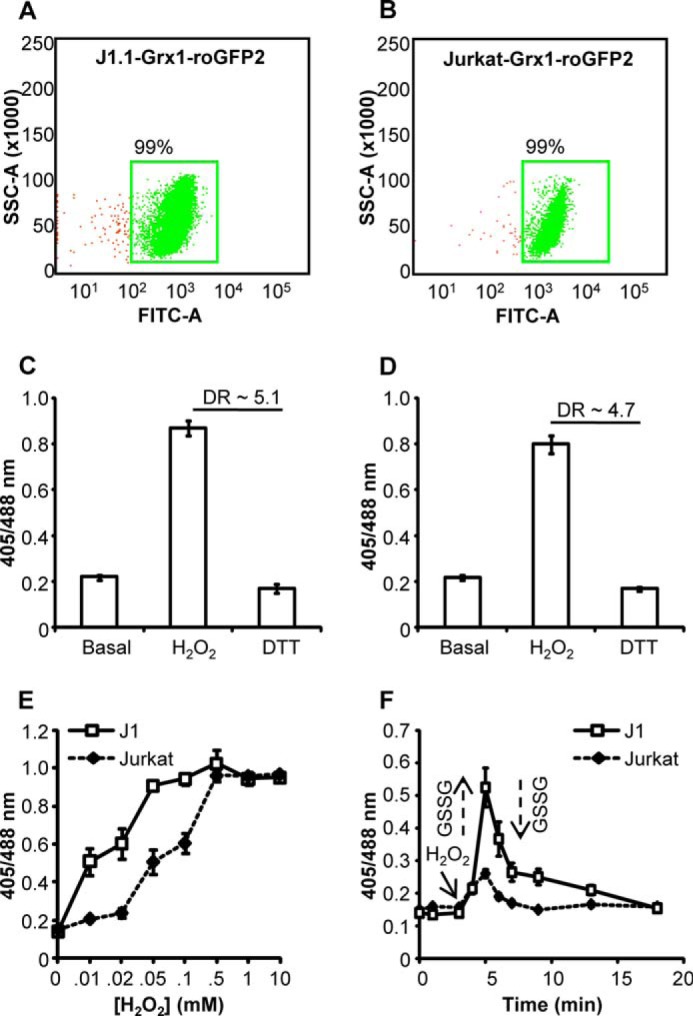
**Generation of J1.1 and Jurkat cells stably expressing the bioprobe and their redox responses toward H_2_O_2_.** Dot plot of J1.1 cells (*A*) and Jurkat cells (*B*) expressing the biosensor in the cytosol is shown. Response of J1.1 cells (*C*) and Jurkat cells (*D*) to exogenously applied oxidant H_2_O_2_ (10 mm) and reductant DTT (10 mm) for 5 min is shown. *DR,* dynamic range. *E,* J1.1 and Jurkat cells expressing cytosolic *E*_GSH_ probe were treated with varying concentrations of H_2_O_2_ for 2 min, and ratiometric sensor response was measured. *F,* J1.1 and Jurkat cells expressing cytosolic biosensor were treated with 10 μm H_2_O_2_, and the ratiometric sensor response was measured over time. *Arrows* represent increase or decrease in subcellular GSSG pool in response to oxidative or anti-oxidative response, respectively. *Error bars* represent S.D. from the mean (*n* = 3). *RFU*, relative fluorescence unit.

Finally, to examine whether the oxidative stress-induced dynamic change in the *E*_GSH_ of U1 is not a cell type-specific phenomena, experiments were carried out using the lymphoid Jurkat T-cell line (J1.1) latently infected with HIV-1 (30). We stably expressed the cytosolic *E*_GSH_ bioprobe in J 1.1 as explained earlier. As a control, we expressed Grx1-roGFP2 in the uninfected Jurkat cells. Expression of Grx1-roGFP2 in J1.1 and Jurkat cells was confirmed by flow cytometer (Fig. 3, *A* and *B*). Similar to U1/U937 cells, Grx1-roGFP2 demonstrated a dynamic range of ∼5.1 in J1.1 cells (Fig. 3*C*) and ∼4.7 in Jurkat cells (Fig. 3*D*). Furthermore, basal *E*_GSH_ of Jurkat and J1.1 cells was also comparable with U1/U937 cells (*i.e.* −309 ± 2 mV). Next, we exposed J1.1 and Jurkat cells to increasing concentrations of H_2_O_2_ for 2 min and measured changes in *E*_GSH_. Similar to U1, H_2_O_2_-induced biosensor oxidation was significantly higher in the case of J1.1 as compared with Jurkat cells (Fig. 3*E*). Similarly, time kinetics of biosensor oxidation-reduction in J1.1 upon treatment with a low concentration of H_2_O_2_ (10 μm) also revealed a significantly greater oxidation of Grx1-roGFP2 as compared with Jurkat cells (Fig. 3*F*). Together, our results indicate that the GSH/GSSG redox couple in persistently infected cells displayed a high degree of responsiveness toward oxidative challenge as compared with uninfected counterparts.

##### U1 Cells Accumulate Lower Levels of Endogenous ROS and Resist Apoptosis upon Oxidative Stress

**FIGURE 4.**
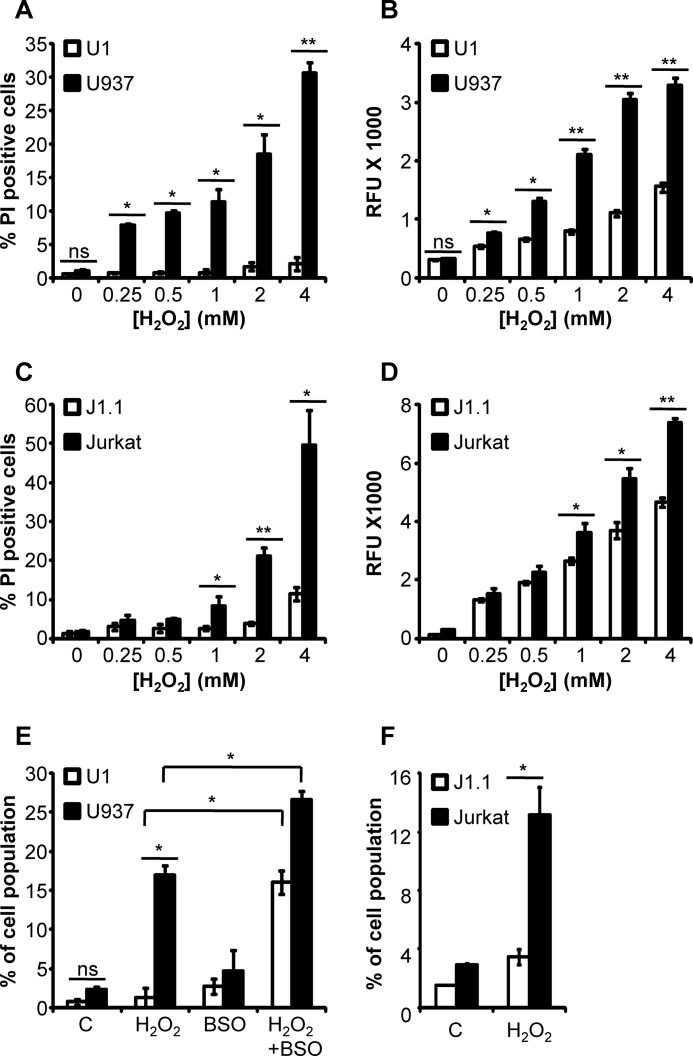
**U1 and J1.1 cells are intrinsically resistant to oxidative stress and apoptosis.** *A,* U1 and U937 cells were treated with different concentrations of H_2_O_2_ for 4 h and stained with PI. *B,* U1 and U937 cells, loaded with CMH_2_DCF-DA, were treated with increasing concentrations of H_2_O_2_, and DCF fluorescence was assessed by flow cytometry. PI staining (*C*) and ROS detection (*D*) in J1.1 and Jurkat cells upon H_2_O_2_ treatment are shown. An increase in the DCF fluorescence reflects the increase in ROS levels upon H_2_O_2_ treatment. *E,* U1 and U937 cells were treated with 50 μm H_2_O_2_ in the absence or presence of 250 μm BSO. After 24 h, cells were harvested and analyzed for apoptosis by annexin-V/PI staining. *F,* apoptosis in J1.1 and Jurkat cells upon treatment with 10 μm H_2_O_2_ for 24 h. The percentages of annexin-V^+^/PI^−^ cells are shown. *C* represents untreated control. *Error bars* represent S.D. from the mean (*n* = 3). *, *p* < 0.01; **, *p* < 0.001, *ns,* not significant.

We next analyzed the consequence of variations in *E*_GSH_ of U1 and U937 cells on intracellular ROS levels and cell death upon H_2_O_2_ exposure. The Grx1-roGFP2 nonexpressing U937 and U1 cells were treated with different concentrations of H_2_O_2_ and stained with CMH_2_DCFDA (for ROS detection) and PI (for necrotic death). The CMH_2_DCFDA is a cell-permeable compound that is oxidized by peroxynitrite, hydroxyl radical, and other ROS to generate an easily detectable fluorescent derivative (31). We found that H_2_O_2_ treatment induced a higher frequency of PI-positive cells and accumulated higher levels of endogenous ROS in U937 cells as compared with U1 at each of the tested concentrations (Fig. 4, *A* and *B*). As an additional verification, we measured ROS levels and oxidative stress resistance phenotype of J1.1 and Jurkat cells upon H_2_O_2_ treatment. As shown in Fig. 4, *C* and *D*, Jurkat cells showed a higher frequency of PI-positive fractions and accumulated elevated ROS levels upon H_2_O_2_ challenge as compared with J1.1 cells.

It is believed that latently infected macrophages are stable viral reservoirs due to their ability to resist apoptosis and cell death (32, 33). We reasoned that rapid and dynamic changes in *E*_GSH_ might be one of the signaling events regulating susceptibility to oxidant-induced apoptosis in U1 cells. To investigate this, we treated U1 and U937 cells with 50 μm H_2_O_2_ for 24 h, and apoptosis was evaluated with annexin V/PI staining. Exposure of cells to low concentrations of H_2_O_2_ for 24 h is known to induce apoptosis in mammalian cells via increasing intracellular ROS generation (34). Treatment with H_2_O_2_ induced annexin-V^+^/PI^−^ profile in 17% of uninfected U937 cells, whereas a negligible fraction (∼2%) of U1 cells showed annexin-V^+^/PI^−^ profile (*p* < 0.01) (Fig. 4*E*). To understand the contribution of *E*_GSH_ in apoptotic resistance during HIV-1 latency, we treated U1 and U937 cells with a nontoxic dose of BSO (250 μm) and monitored H_2_O_2_-induced apoptosis. Although BSO alone does not induce significant apoptosis (Fig. 4*E*), co-treatment with BSO and H_2_O_2_ reversed the resistant phenotype of U1 and led to a potent induction of apoptosis in these cells. The frequency of annexin-V^+^/PI^−^ cells increased in both cell types after BSO treatment (Fig. 4*E*). Similar to U1 cells, 20 μm H_2_O_2_ treatment induced the annexin-V^+^/PI^−^ profile in 14% of uninfected Jurkat cells as compared with 3% in latently infected J1.1 cells (*p* < 0.01) (Fig. 4*F*). Taken together, data demonstrate that chronic HIV-1 infection is associated with increased redox response and tolerance toward oxidative stress-mediated apoptosis and death.

##### E_GSH_ Modulates HIV-1 Activation

**FIGURE 5.**
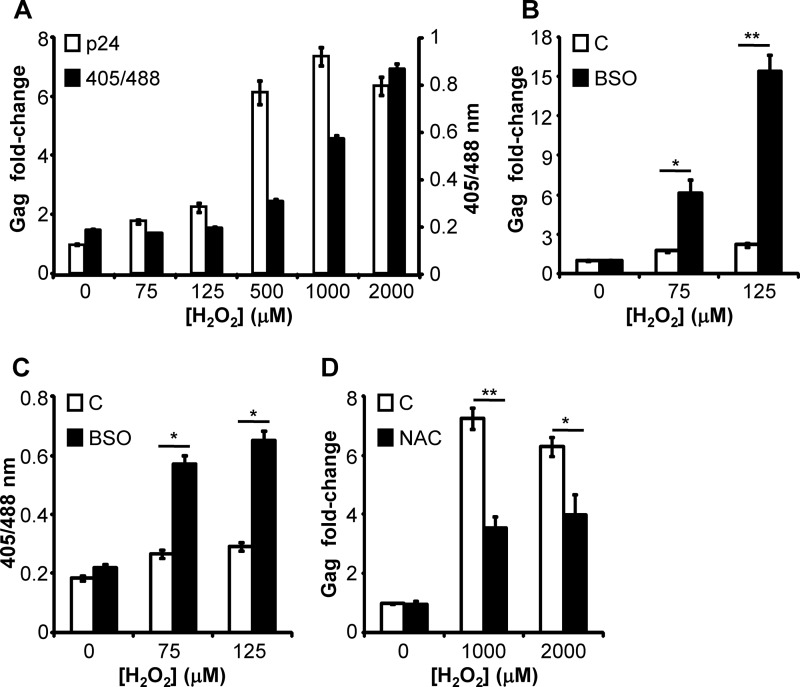
**Effects of *E*_GSH_ on HIV-1 activation.** *A,* U1 cells were exposed to varying concentrations of H_2_O_2_ for 6 h after which cells were harvested and analyzed for viral activation by Gag qRT-PCR. In a parallel experiment, ratiometric sensor response was measured by flow cytometry. *B,* U1 cells were treated with 75 and 125 μm of H_2_O_2_ in the absence or presence of 250 μm BSO. After 6 h, cells were subjected to Gag qRT-PCR. *C,* in a parallel experiment, ratiometric sensor response was measured by flow cytometry. *D,* U1 cells were treated with 15 mm NAC for 2 h followed by exposure to 1 and 2 mm of H_2_O_2_. After 6 h, the extent of viral activation was analyzed by Gag qRT-PCR. *Error bars* represent S.D. from the mean (*n* = 3). *, *p* < 0.01; **, *p* < 0.001; *C*, control.

Increased oxidative stress and decreased intracellular thiols were shown to activate the HIV-1 long terminal repeat (LTR) through the redox-responsive transcription factor NFκB (2, 3). However, the role of intracellular *E*_GSH_ in modulating HIV-1 activation from chronically infected macrophages has never been addressed. Having shown that U1 cells are resilient to H_2_O_2_, we next examined how this affects the ability of H_2_O_2_ to induce HIV-1 activation in these cells. The U1 cells were treated with defined concentrations of H_2_O_2_ to precisely manipulate intracellular *E*_GSH_, and the expression of HIV-1 Gag transcript was monitored as a marker of virus activation by qRT-PCR at 6 h post-treatment. We observed that those concentrations of H_2_O_2_ (75 and 125 μm), which were ineffective in inducing a sustained oxidative shift in *E*_GSH_, also did not induce HIV-1 transcription (Fig. 5*A*). However, a modest but stable increase in intracellular *E*_GSH_ (approximately −285 mV) upon treatment with 0.5 mm H_2_O_2_ led to a significant induction of Gag expression in U1 cells (Fig. 5*A*). Time points earlier than 6 h (*i.e.* 4 h) failed to show any significant stimulation of Gag transcription by H_2_O_2_ (data not shown), thus confirming that we are capturing early induction in HIV-1 expression caused by a change in cellular and subcellular *E*_GSH_. These results suggest that the inherent capacity of U1 cells to neutralize oxidative stress via the GSH pathway confers insensitivity toward oxidant-mediated activation of HIV-1 at lower concentrations of H_2_O_2_. To confirm this, we co-treated U1 cells with nonactivating concentrations of H_2_O_2_ and a nontoxic dose of 250 μm BSO (to decrease GSH synthesis), and induction of Gag expression was measured. Strikingly, for nonactivating concentrations of H_2_O_2_, co-treatment with BSO in U1 cells led to a potent Gag transcription (∼5–15-fold) as compared with untreated cells (*p* < 0.01) (Fig. 5*B*). To confirm that the influence of BSO on HIV-1 transcription was due to a shift in *E*_GSH_, we measured intracellular *E*_GSH_ of U1 cells upon co-treatment with BSO and H_2_O_2_. As expected, co-treatment with BSO and H_2_O_2_ (75 and 125 μm) led to sustained oxidation of Grx1-roGFP2 as compared with treatment with BSO or H_2_O_2_ alone (Fig. 5*C*). We reasoned that if oxidized *E*_GSH_ induces virus reactivation, treatment of U1 cells with the GSH-specific antioxidant NAC should reverse this effect. To rule out the effect of NAC on virus activation by directly scavenging H_2_O_2_, we pretreated U1 cells with NAC for 2 h, followed by exposure to 1 and 2 mm H_2_O_2_ and Gag transcript analysis. Pretreatment of U1 cells with NAC effectively blocked viral activation (Fig. 5*D*), suggesting that intracellular *E*_GSH_ is important for controlling HIV-1 persistence and activation. Importantly, these findings reveal the potential of redox-oriented compounds for modulating the persistence and reactivation of HIV.

##### Activation of HIV-1 Induces a Sustained Oxidative Shift in Intracellular E_GSH_

In addition to the role of ROS in reactivating HIV-1, active replication of HIV-1 is also known to trigger oxidative stress in the host (12, 35). The HIV-1 infection-induced oxidative stress eventually results in serious immunological and metabolic complications leading to cellular abnormalities and host death (1). Therefore, real time measurement of *E*_GSH_ following HIV-1 infection can lead to a better understanding of disease progression.

**FIGURE 6.**
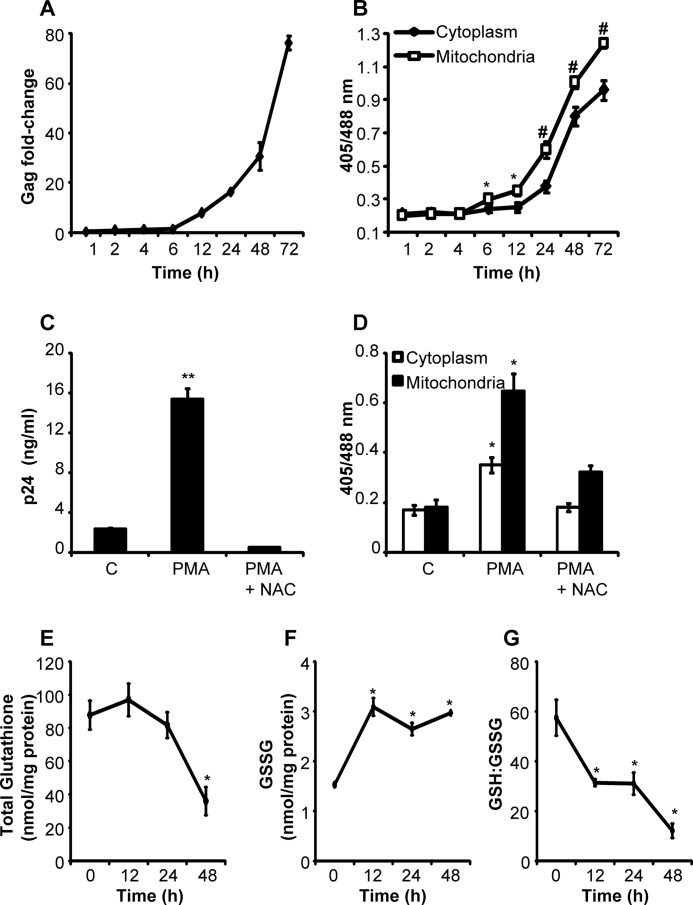
**HIV-1-induced changes in *E*_GSH_ of U1 cells.** *A,* course of HIV-1 replication after stimulation of U1 cells with 5 ng/ml PMA. Viral load was monitored by Gag qRT-PCR. *B,* U1 cells expressing cytosolic and mitochondrial *E*_GSH_ probe were treated with 5 ng/ml PMA, and sensor response was recorded at indicated time points. *p* values shown were calculated by comparing cytosolic and mitochondrial sensor response. U1 cells were treated with PMA in the absence or presence of 15 mm NAC. After 24 h, HIV-1 replication was monitored by p24 ELISA of culture supernatants (*C*), and U1 ratiometric sensor response was monitored by flow cytometry (*D*). *p* values were obtained by comparing samples treated with PMA and PMA + NAC. Whole-cell total glutathione (*E*) and GSSG (*F*) concentrations measured in lysates prepared from PMA-treated U1 cells at indicated time points. *G,* GSH/GSSG ratios were derived from the total glutathione and GSSG values obtained in *E* and *F. p* values are calculated by comparing PMA-treated samples with untreated control. *Error bars* represent S.D. from the mean. All experiments are performed at least twice. *, *p* < 0.05; **, *p* < 0.001; #, *p* < 0.005. *C,* untreated control.

To examine whether HIV-1 replication is associated with a change in *E*_GSH_ of cytosol and/or mitochondria, we first induced HIV-1 expression using a low concentration of PMA (5 ng/ml), an established activator of HIV-1 in U1 cells (36). The U1 cells were found to start expressing detectable levels of HIV-1 Gag transcript as early as 6–12 h post-PMA treatment, which continued to increase for the entire 72 h duration of the experiment (Fig. 6*A*). We then asked whether virus activation correlated with the induction of oxidative shift in *E*_GSH_ of the subcellular compartment of U1 cells. Consistent with this, PMA activation significantly induced oxidation of Grx1-roGFP2 in both cytosol and mitochondria of U1 in a time-dependent manner (Fig. 6*B*). However, compartment-specific differences were also evident. For example, mitochondria displayed an early response (6 h) and higher degree of oxidative shift in *E*_GSH_ in response to PMA treatment, whereas cytosolic *E*_GSH_ showed oxidative deflection at 24 h; the oxidative stress in cytoplasm remained lower than mitochondria throughout the duration of the experiment (Fig. 6*B*). Importantly, the emergence of oxidative stress in mitochondria closely coincided with the initiation of the HIV-1 Gag expression, indicating a better correlation between mitochondrial *E*_GSH_ and viral transcription (Fig. 6, *A* and *B*). Stimulation of U937 transgenic cells with an equivalent concentration of PMA alone does not induce Grx1-roGFP2 oxidation (data not shown), confirming that oxidative stress in U1 cells is mediated by HIV-1 activation. In agreement with this, we show that treatment with NAC completely abrogated PMA-stimulated HIV-1 transcription and mitigated the oxidative shift in *E*_GSH_ of U1 subcompartments (Fig. 6, *C* and *D*).

Additionally, chemical measurements revealed time-dependent variations in whole-cell glutathione content and GSSG concentration upon HIV-1 activation in U1 cells. At 12 and 24 h post-PMA treatment, we did not observe any significant changes (*p* = 0.3) in whole-cell glutathione. However, a significant (*p* < 0.02) decline was observed at 48 h post-PMA treatment (Fig. 6*E*). In contrast, whole-cell GSSG showed a significant (*p* < 0.01) increase at 12 h post-PMA treatment following which no further increase was observed (Fig. 6*F*). Consequently, a significant time-dependent decrease in GSH/GSSG ratio was observed upon HIV-1 replication (Fig. 6*G*). These results indicate that the moderate oxidative shift in *E*_GSH_ observed during early stages of viral replication was likely the consequence of GSH oxidation to GSSG, whereas higher HIV-1 replication resulted in both oxidation and depletion of GSH to induce an overwhelming oxidative shift in *E*_GSH_.

**FIGURE 7.**
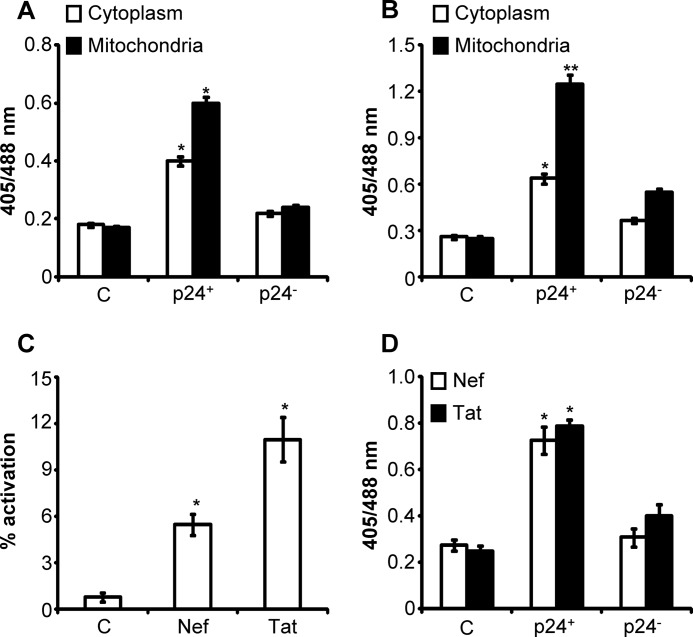
**Diverse HIV-1-activating agents commonly induce oxidative shift in cytosolic and mitochondrial *E*_GSH_ of U1 cells.** U1-Grx1-roGFP2 and U1-mito-Grx1-roGFP2 cells were treated with either 5 ng/ml PMA for 12 h (*A*) or 100 ng/ml TNF-α for 72 h (*B*) followed by intracellular staining for HIV-1 p24. *E*_GSH_ of p24^+^ and p24^−^ cells was determined using multi-parameter flow cytometric analysis. Percent HIV-1 activation (*C*) and *E*_GSH_ of p24^+^ and p24^−^ U1 cells (*D*) following treatment with Nef and Tat proteins for 72 h were determined by intracellular staining for HIV-1 p24 and multiparameter flow cytometric analysis, respectively. *p* values were calculated by comparing treated cells with untreated controls in each panel. *C,* untreated control. *Error bars* represent S.D. from the mean (*n* = 3). *, *p* < 0.01; **, *p* < 0.001.

Exposure to PMA induces phenotypic heterogeneity in U937 cells (37), which can induce variable degrees of HIV-1 activation to distinctly modulate *E*_GSH_ in these cells. Therefore, to rule out the influence of this variation on our measurements, we stained U1 cells 12 h post-PMA treatment with antibodies against HIV-1 p24 antigen, and we measured the cytosolic and mitochondrial *E*_GSH_ of p24^+^ (active replication) and p24^−^ (latently infected) cells by multiparametric flow cytometry. Significant levels of oxidative stress were only evident in p24^+^ U1 cells, whereas p24^−^ cells showed only background response (Fig. 7*A*). In agreement with the earlier results, mitochondria demonstrated greater shifts in *E*_GSH_ as compared with cytosolic fractions of p24^+^ U1 cells (Fig. 7*A*). Because cellular cytokines also stimulate HIV-1 transcription (26), as an additional verification of HIV-1 expression-induced oxidative stress, the effect of TNF-α mediated HIV-1 activation on this oxidative shift in *E*_GSH_ was examined. As shown in Fig. 7*B*, TNF-α induced a substantial oxidative shift in p24^+^ cells as compared with untreated or p24^−^ cells. The HIV accessory proteins Tat and Nef are known to activate virus expression (38); we therefore checked whether the exogenous addition of physiologically relevant concentrations (100 ng/ml) of Tat or Nef activates HIV-1 to induce oxidative stress. We found that both these viral proteins simultaneously induce HIV-1 activation and oxidative shift in *E*_GSH_ of U1 cells (Fig. 7, *C* and *D*).

**FIGURE 8.**
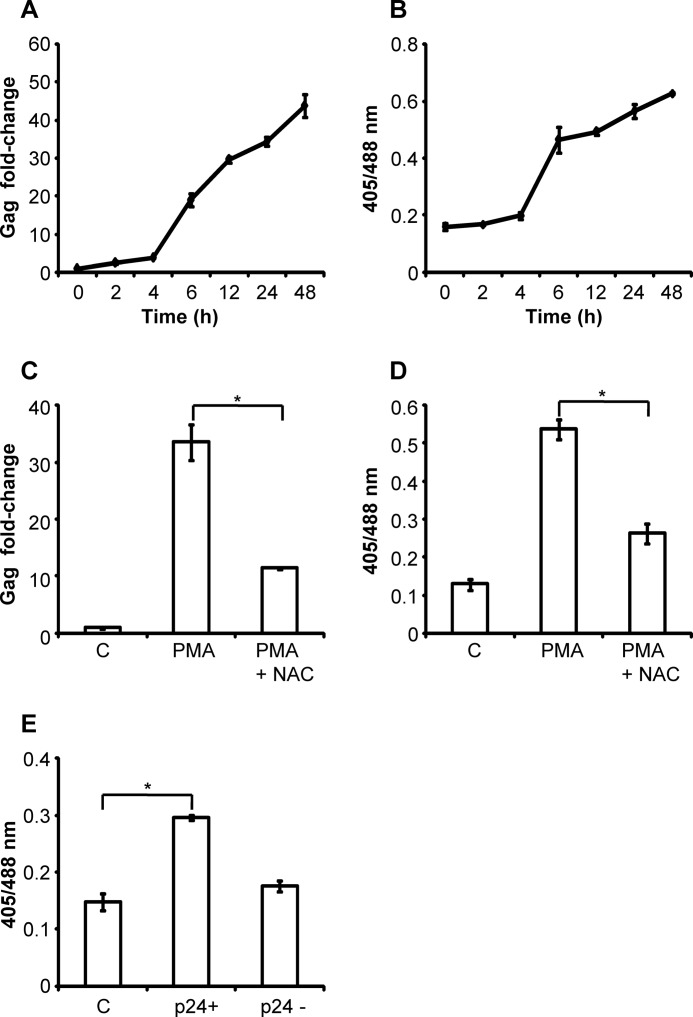
**HIV-1-induced changes in *E*_GSH_ of J1.1 cells.** Viral load (*A*) and biosensor response (*B*) in J1.1 cells after stimulation with 5 ng/ml PMA are shown. J1.1 cells were treated with PMA in the absence or presence of 5 mm NAC (*C*). After 24 h, HIV-1 replication was monitored by Gag qRT-PCR. In a parallel experiment, biosensor response was monitored by flow cytometry (*D*). *E, E*_GSH_ of p24^+^ and p24^−^ J1.1 cells after treatment with 100 ng/ml TNF-α for 72 h. *Error bars* represent S.D. from the mean. *, *p* < 0.05. *C,* untreated control.

Using J1.1 cells, we provided further verification of HIV-1 replication-mediated oxidative shift in *E*_GSH_ in a time-dependent fashion (Fig. 8, *A* and *B*). Consistent with U1 data, treatment with NAC abrogated PMA-induced HIV-1 transcription and reversed Grx1-roGFP2 oxidation in J1.1 (Fig. 8, *C* and *D*). Finally, we activated HIV-1 from J1.1 cells by treating with TNF-α and measured the changes in *E*_GSH_. Similar to U1 cells, a substantial oxidative shift was only observed in p24^+^ J1.1 cells as compared with untreated or p24^−^ cells (Fig. 8*E*). Taken together, using several different activation strategies, we demonstrate that HIV-1 expression perturbs glutathione redox homeostasis in infected cells.

##### M. tuberculosis Infection Modulates E_GSH_ to Induce HIV-1 Activation

**FIGURE 9.**
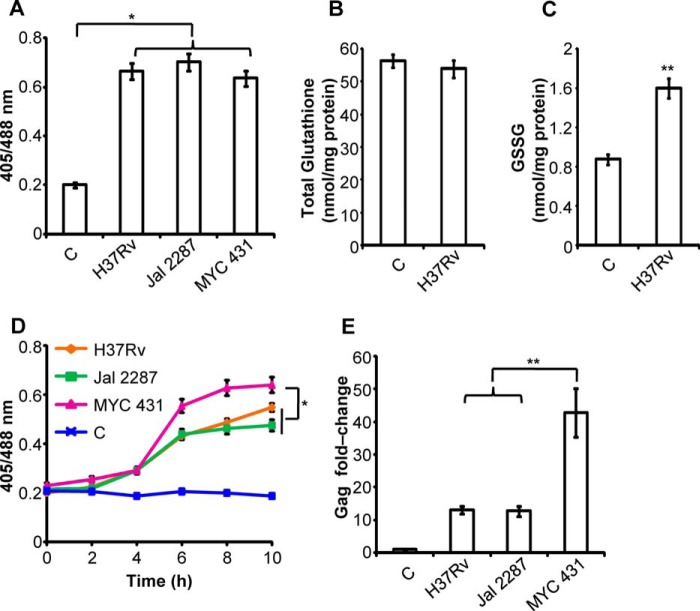
***M. tuberculosis* polyketide lipids induce substantial oxidative shift in *E*_GSH_ to activate HIV-1 from persistence.** *A,* U937-Grx1-roGFP2 cells were infected with *M. tuberculosis* strains H37Rv, Jal 2261, and MYC 431 at a multiplicity of infection of 10. 48 h post-infection, cells were treated with NEM-PFA, and sensor response was measured by flow cytometry. Whole-cell total glutathione (*B*) and GSSG (*C*) concentrations measured in lysates prepared from U937 cells uninfected or infected with H37Rv for 48 h. *D,* time course of biosensor oxidation in U937-Grx1-roGFP2 cells exposed to 50 μg surface-exposed lipids extracted from different *M. tuberculosis* strains. *p* values were calculated by one-way analysis of variance followed by Tukey's HSD statistical test. *E,* Gag RT-PCR of U1 cells treated with 50 μg/ml surface-exposed lipids extracted from *M. tuberculosis* strains H37Rv, Jal2261, and MYC 431 for 48 h. *C,* uninfected control in *A–C*, and untreated control in *D* and *E. Error bars* represent S.D. from the mean. All experiments are performed at least twice. *, *p* < 0.05; **, *p* < 0.01; *C*, control.

Co-infection with *M. tuberculosis* is one of the leading causes of death due to HIV-1 infection (39). Furthermore, HIV-1-infected individuals are at a greater risk of acquiring tuberculosis infection by the emerging drug-resistant *M. tuberculosis* strains (40). Because *M. tuberculosis*-infected individuals and experimental animal models show depleted levels of GSH (41, 42), we hypothesized that *M. tuberculosis* could activate HIV-1 from latency in U1 cells by inducing an oxidative shift in *E*_GSH_. To investigate this, we used one virulent laboratory strain (H37Rv) and two drug-resistant Indian clinical strains (multidrug-resistant Jal 2287 and extensively drug-resistant MYC 431) of *M. tuberculosis* (43). An experimental analysis involving macrophages infected with a BSL3 class pathogen such as *M. tuberculosis* requires chemical fixation by PFA, which is well known to oxidize roGFP2-based biosensors (19, 21, 43). To circumvent PFA-mediated oxidation artifact, we alkylated the thiols of Grx1-roGFP2 using the cell-permeable fast-acting thiol modifier NEM before PFA fixation and FACS analysis. NEM treatment is known to effectively prevent PFA-mediated oxidation of roGFP2 biosensor in various studies performed on diverse cells and tissues (19, 21, 43). With this chemical fixation strategy in hand, we checked the ability of *M. tuberculosis* strains to induce a change in *E*_GSH_ of U937 monocytes by infecting these cells harboring cytosolic Grx1-roGFP2 at a multiplicity of infection of 10 (10 bacteria/macrophage) and measuring *E*_GSH_ at 48 h post-infection. As shown in Fig. 9*A*, all three *M. tuberculosis* strains induced substantial oxidative shift in *E*_GSH_ of U937 cytosol. We also measured whole-cell glutathione and GSSG levels in U937 cells infected with H37Rv. As is shown in Fig. 9, *B* and *C*, whole-cell glutathione was not significantly (*p* = 0.4) affected by H37Rv infection; however, whole-cell GSSG was significantly increased (*p* < 0.01) (Fig. 9*C*). These results indicate that biosensor oxidation was mediated by the oxidation of GSH to GSSG in response to *M. tuberculosis* infection.

Because HIV-1 infects multiple cell types, including macrophages, dendritic cells, lymphocytes, etc., whereas *M. tuberculosis* mainly resides inside macrophages, one way by which *M. tuberculosis* uniformly modulates redox physiology of diverse HIV-infected cells is by producing secreted virulence factors. In this context, *M. tuberculosis* produces complex cell wall-associated secretory virulence lipids/polyketides, which are known to modulate the host innate immune response (24). We therefore analyzed the oxidizing potential of *M. tuberculosis* lipids in U937 cells. The *M. tuberculosis* surface-exposed lipids were extracted; U937-Grx1-roGFP2 cells were treated with 50 μg of lipids, and at various time points redox measurements were performed. We found that *M. tuberculosis* lipids were highly active in inducing Grx1-roGFP2 oxidation inside U937 cells (Fig. 9*D*). Interestingly, lipids isolated from the XDR strain, MYC 431, were found to be most effective in generating oxidized *E*_GSH_ in U937 cells (Fig. 9*D*). These results confirm that *M. tuberculosis* strains are potent inducers of oxidative stress in macrophages and that bioactive lipids are one of the mediators of this effect.

Taking insight from these findings, we investigated whether *M. tuberculosis* lipids activate HIV-1 from latency. We treated HIV-1-infected U1 cells with the total lipids derived from *M. tuberculosis* strains, and HIV-1 activation was analyzed by quantifying Gag mRNA levels. The exposure of *M. tuberculosis* lipids led to a strain- and stress-dependent increase in the Gag expression (Fig. 9*E*). In line with earlier results, the greater oxidizing potential shown by MYC 431 lipids also translated into a significantly higher degree of HIV-1 activation (Fig. 9*E*). Taken together, we have identified a previously uncharacterized role of *M. tuberculosis* lipids in modulating *E*_GSH_ and inducing the HIV-1 expression in chronically infected macrophages and established the utility of Grx1-roGFP2 in dissecting redox signaling mechanisms associated with HIV and HIV-*M. tuberculosis* co-infection.

##### HIV-1 Latency and Reactivation Modulates Expression of Oxidative Stress-related Gene Expression Profile

**FIGURE 10.**
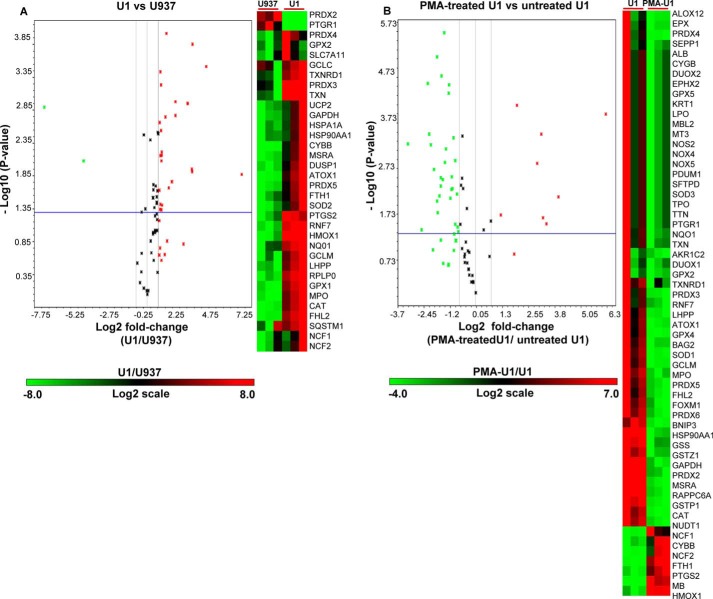
**Expression of oxidative stress-responsive genes during HIV-1 latency.** Total RNA was isolated from untreated U937, untreated U1, and PMA-treated U1 cells followed by expression analysis of 84 genes specific to oxidative stress pathways (SABiosciences qRT-PCR array profiler). Oxidative stress response-specific gene expression profiles were compared in the following groups: *A,* untreated U1 *versus* untreated U937; *B,* PMA-treated U1 *versus* untreated U1. Shown are the volcano plots displaying statistical significance *versus* fold-change on the *y* and *x* axes, respectively. *Horizontal blue line* shows the cutoff of *p* value (0.05) and *vertical black lines* depict cutoff levels for gene expression (2-fold variation). Also shown are the heat maps of differentially regulated genes in three biological replicates. The *color bar* depicts the level of gene expression. The data are the mean of three independent experiments.

Having shown the importance of *E*_GSH_ in HIV-1 latency and reactivation, we next wanted to validate our findings using alternative redox signaling technologies. To this end, we performed oxidative stress and antioxidant defense pathway-focused human gene expression array (RT^2^ Profiler^TM^ PCR arrays SABiosciences, Frederick, MD) during the latent and reactivation phase of HIV-1 infection. Because latently infected U1 monocytes demonstrated lower susceptibility to oxidative stress and apoptosis as compared with uninfected U937 cells, we first conducted PCR array analysis on these cells to further understand the redox basis of latency maintenance. Analysis of a PCR array containing 84 genes related to oxidative stress demonstrates differential regulation of 34 genes in U1 as compared with U937 (Fig. 10*A* and supplemental Table S1). Interestingly, more than 90% of genes showing altered expression were up-regulated in U1 cells. Of these, 25 genes encoding common antioxidants were up-regulated 2–4-fold as compared with U937 cells. This included catalase, superoxide dismutase, peroxiredoxin family, glutathione peroxidases (GPXs), glutamate-cysteine ligase, etc. Some of the highly expressed oxidative stress-responsive genes included peroxiredoxin-3 (PRDX3; 8-fold), thioredoxin-reductase (TXNRD1; 9-fold), cytochrome *b*_245_ (91-fold), heme oxygenase (decycling) 1 (HMOX1; 9-fold), and myeloperoxidase (7-fold). The increased expression of antioxidants seems to be consistent with the Grx1-roGFP2 results showing superior capacity of U1 to respond to and mitigate oxidative stress as compared with U937.

**FIGURE 11.**
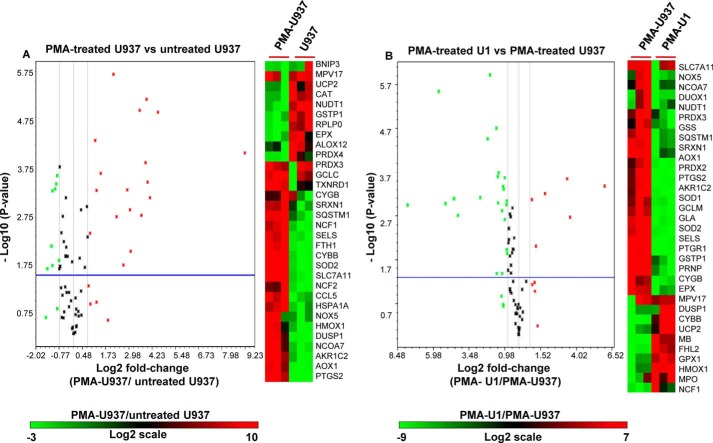
**Expression of oxidative stress responsive genes during reactivation.** Total RNA was isolated from untreated U937, PMA-treated U937, and PMA-treated U1 cells followed by expression analysis of 84 genes specific to oxidative stress pathways (SABiosciences qRT-PCR array profiler). *A,* PMA-treated U937 *versus* untreated U937; *B,* PMA-treated U1 *versus* PMA-treated U937. Shown are the volcano plots displaying statistical significance *versus* fold-change on the *y* and *x* axes, respectively. *Horizontal blue line* shows the cutoff of *p* value (0.05) and *vertical black lines* depict cutoff levels for gene expression (2-fold variation). Also shown are the heat maps of differentially regulated genes in three biological replicates. The *color bar* depicts the level of gene expression.

Because our redox bioprobe demonstrated an oxidative shift in *E*_GSH_ upon activation of the viral replication cycle, we next studied how the expression pattern shown by U1 is altered upon HIV-1 induction. As shown earlier, we observed that PMA-mediated differentiation of U1 cells triggers viral replication in a time-dependent manner (Fig. 6*A*). By 48 h post-induction, we observed high levels of Gag transcript but with increased cell death due to cytopathic effects (data not shown). To ensure that changes in gene expression are due to viral replication and not because of cell death due to HIV-1 replication, we performed expression analysis at 24 h post-induction. At this time, the viability of induced U1 cells remained at levels comparable with that of PMA-treated uninfected U937 cells (data not shown). Both U1 and U937 cells were subjected to identical PMA treatments, and isolated RNA samples were subjected to PCR arrays as described earlier. We observed that PMA treatment of U1 down-regulated a majority of stress-responsive genes as compared with untreated U1 (Fig. 10*B* and supplemental Table S1). Interestingly, an opposite trend was observed in the case of PMA-treated U937 cells compared with untreated cells (Fig. 11A and supplemental Table S1), indicating that the down-regulation of expression observed in the case of U1 is a consequence of viral replication rather than PMA treatment. Direct comparison of expression profiles upon PMA treatment revealed altered regulation of 36 genes in U1 cells as compared with U937 cells (Fig. 11*B* and supplemental Table S1). As expected, expression profiles indicate the prevalence of pro-oxidizing conditions during HIV-1 induction and replication. For example, whereas key cellular antioxidants such as superoxide dismutase (SOD1 and SOD2), glutathione synthetase (GSS), glutathione transferase P1 (GSTP1), glutamate-cysteine ligase, peroxiredoxins, sequestosome 1 (SQSTM1), and sulfiredoxin (SRXN1) were down-regulated, genes involved in increasing cellular ROS levels (*e.g.* NADPH oxidase component (cytochrome *b*_245_) and mitochondrial uncoupling protein (UCP2)) were induced (44, 45). In addition, several genes directly involved in modulating ROS levels were altered. This included arachidonate 12-lipoxygenase (ALOX12) (46), aldehyde oxidase 1 (AOX1) (47), dual oxidase 1 (DUOX1) (48), NADPH oxidase (NOX5) (44), prostaglandin-endoperoxide synthase 2 (PTGS2), prostaglandin reductase 1 (PTGR1) (49), etc. (Fig. 11*B* and supplemental Table S1).

**FIGURE 12.**
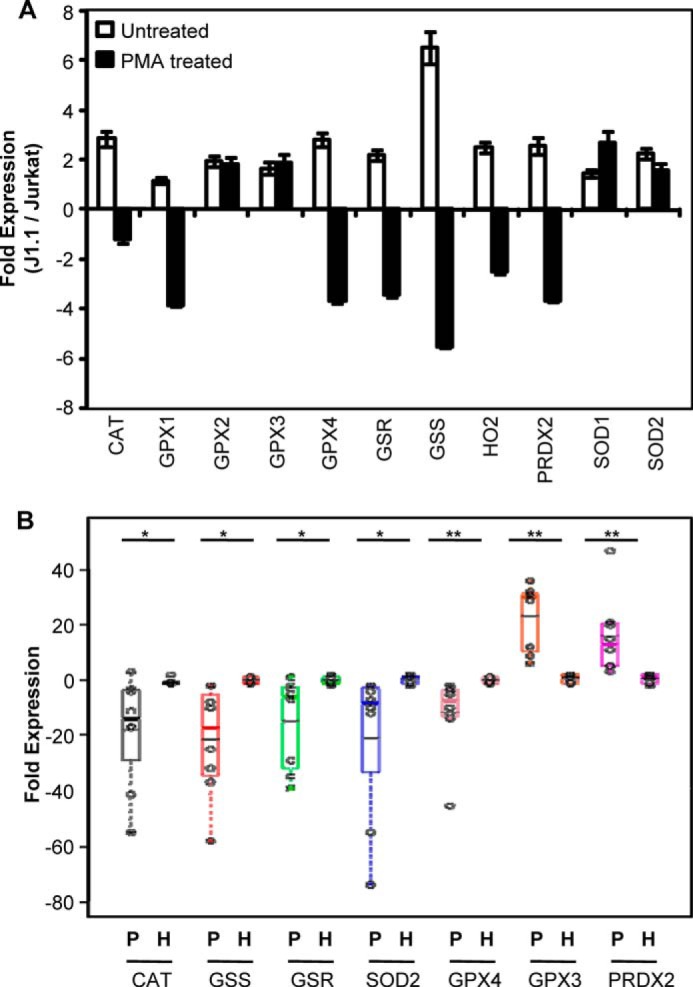
*A,* expression of antioxidant genes in J1.1 cells during latency and reactivation. Total RNA was isolated from untreated and PMA-treated Jurkat and J1.1 cells followed by qRT-PCR analysis of oxidative stress response-related gene expression. Shown is the fold change in gene expression in J1.1 as compared with Jurkat cells. Human β-actin is used as an internal control. The data are the mean of three independent experiments ±S.D. *B,* expression of oxidative stress genes in the PBMCs of HIV/AIDS patients. qRT-PCR analysis of oxidative stress response-related gene expression in PBMCs derived from HIV/AIDS patients (*P*, *n* = 8) and healthy individuals (*H*, *n* = 6). One of the healthy individuals was taken as the reference point, and human β-actin was used as an internal control. Data are expressed as a dot plot with the median, and mean and significant values are indicated on the top. Each *dot* on the plot represents an individual. *, *p* < 0.01; **, *p* < 0.001; *CAT*, catalase.

In agreement with U1/U937 expression profile, J1.1 cells demonstrated a consistent increase in several antioxidant genes as compared with Jurkat cells. Furthermore, PMA-mediated activation of HIV-1 and subsequent HIV replication led to significant down-regulation of antioxidant expression in J1.1 as compared with Jurkat cells (Fig. 12*A*).

To validate the physiological relevance of our cell line-based findings, we performed qRT-PCR analysis of a selected set of oxidative stress genes on RNA isolated from the PBMCs of HIV-1-infected symptomatic individuals. From a total of 11 genes selected for this analysis, seven genes showed differential expression (Fig. 12*B*), out of which five were significantly down-regulated in HIV patients as compared with healthy individuals. The genes significantly down-regulated in HIV-1 patient PBMCs including *CAT*, *GSR, GSS, GPX4,* and *SOD2* are well characterized for their role in neutralizing oxidative stress.

## DISCUSSION

Fundamental research in the area of HIV-1 basic biology is hampered by the lack of tools to accurately probe the physiology of HIV-1-infected cells during various stages of infection and drug therapy. Because oxidative stress and glutathione appear to play an important role in HIV infection (11), it is important to provide a correct numerical assessment of intracellular *E*_GSH_ of HIV-1-infected immune cells. Here, we applied a noninvasive biosensor, Grx1-roGFP2, to image dynamic changes in the *E*_GSH_ of HIV-1-infected cells. The basal cytosolic and mitochondrial *E*_GSH_ of U937, U1, Jurkat, and J1.1 cells are comparable but highly reduced (approximately −310 mV). Considering that previous estimates reported a relatively oxidized *E*_GSH_ (approximately −240 mV) in diverse mammalian cells (7), our findings suggest that this discrepancy could be due to the following: (*a*) loss of glutathione; (*b*) oxidation of glutathione; or (*c*) mixing glutathione from various subcompartments due to the use of cell-disruptive methodologies in the earlier studies. In support of this, we performed enzymatic analysis of the GSH/GSSG ratio using whole-cell extract of U1 and U937 cells and confirmed a ratio of ∼60:1, which is equivalent to *E*_GSH_ approximately −220 mV, and is in good agreement with the previous studies (7). Furthermore, our findings are in line with recent estimates of cytosolic *E*_GSH_ generated using roGFP2 in other mammalian cells and organisms such as HeLa cells (−325 mV) (29), *Saccharomyces cerevisiae* (−320 mV) (18), *Arabidopsis thaliana* (−318 mV) (28), and *Drosophila* (−320 mV) (21).

Using Grx1-roGFP2, we demonstrated dynamic changes in cytosolic and mitochondrial *E*_GSH_ of U1 and U937 cells upon oxidative challenge. In contrast, whole-cell GSSG analyses revealed continued oxidative *E*_GSH_ in these cells. This inconsistency with conventional measurements can only be explained if GSSG formed in response to oxidative stress is rapidly removed from the cytosol and mitochondria to other cellular compartments as a mechanism to maintain cytosolic/mitochondrial glutathione redox homeostasis. Consistent with this view, a recent study in yeast demonstrated that rapid transport of cytosolic GSSG to vacuoles is the main mechanism that restores cytosolic *E*_GSH_ balance upon an oxidative insult (50). Our findings suggest a similar mechanism underlying the maintenance of glutathione redox homeostasis in monocytes upon oxidative stress and propose that future strategies should dissect the role of pathways involved in GSSG removal, reduction, and sequestration in the context of HIV-1 infection.

Having shown that cytosolic and mitochondrial steady-state *E*_GSH_ remained comparable in U1 and U937 monocytes, we next discovered that exposure to physiologically relevant concentrations of H_2_O_2_ led to a substantially higher oxidative shift in *E*_GSH_, increased whole-cell GSSG, and enhanced resistance to apoptosis in U1 as compared with U937 cells. It can be argued that the overexpression of Grx1-roGFP2 can adversely influence cellular physiology to induce variations in the oxidative stress phenotype observed between U937 and U1 monocytes. However, H_2_O_2_ induces a comparable degree of death in biosensor expressing or nonexpressing cells (data not shown). Moreover, J1.1 cells also exhibited a higher oxidative shift in *E*_GSH_ and displayed better resistance toward H_2_O_2_-induced apoptosis as compared with Jurkat cells.

Although oxidative *E*_GSH_ and higher whole-cell GSSGs are generally considered as markers of oxidative stress (7), our findings using U1 and J1.1 cells appeared counter-intuitive. The data suggest that latently infected cells are better equipped to tolerate oxidative stress despite higher whole-cell GSSG and increased oxidative shift in *E*_GSH_ of subcellular compartments in response to the H_2_O_2_ challenge. The explanation may be that in latently infected cells, H_2_O_2_ is predominantly metabolized by endogenous GPXs, which in the process convert GSH to GSSG thereby inducing higher biosensor oxidation. In uninfected cells, GSH-assisted catabolism of H_2_O_2_ by GPXs is either inefficient or H_2_O_2_ is preferentially metabolized by other mechanisms (*e.g.* thioredoxins-coupled peroxidases). This interpretation is strengthened by our findings showing high basal level gene expression of several GPXs in U1 and J1.1 cells as compared with uninfected counterparts. In this context, it is interesting to note that a homolog of human GPX is also encoded by HIV-1 (51), which protects infected cells from ROS-mediated apoptosis. Importantly, a study indicates that the clinical HIV-1 isolates from long term nonprogressors contain an intact and functional GPX, whereas nonfunctional GPX was associated with isolates from AIDS patients (51). Although the contribution of HIV-1 GPX in mediating oxidative stress resistance in U1 and J1.1 is presently unknown, it is possible that basal expression of HIV-1 GPX along with elevated host GPXs jointly assist latently infected cells to neutralize oxidative stress. Finally, enhanced expression of several cellular antioxidants in U1 and J1.1 confirms superior oxidative stress resistance capacity of these cells. Together, our findings highlight the complexities associated with interpreting the presence or absence of oxidative stress and suggest the use of multiple analytical and genetic technologies to comprehensively investigate the oxidative status of a cell.

Our results along with a recent study demonstrating the role of mitochondrial membrane potential in HIV persistence (52) indicate a central role for redox and energy homeostatic pathways in the maintenance of long term reservoirs of HIV-1-infected cells. Our data suggest that effective resistance toward oxidative stress and apoptosis via GSH-mediated redox signaling can be a common phenotype of cells persistently infected with HIV-1. For example, HIV-1 remains latent in astrocytes (53) and in resting memory CD4^+^ T cells (54), which are extraordinarily resilient to oxidative stress and cell death due to mechanisms known to restore GSH redox balance (55–58). Whether this differential regulation of *E*_GSH_ between U1/J1.1 and U937/Jurkat is specific for these cell lines or represents a general difference between uninfected and latently infected cells needs further investigation.

Recently, reactivation of latent viruses coupled with highly active antiretroviral therapy (HAART) has been proposed as a possible “shock and kill” strategy to purge latent reservoirs (59). Based on this approach, a high throughput screening effort identified a ROS generating molecule as an effective activator of latent viruses in CD4^+^ T cells (60). Because ROS can have multiple disturbing influences on the general physiology of a cell, a precise understanding of oxidative potential changes required to activate the virus from latency is central to the success of these paradigmatic approaches. Applying this biosensor, we provide an accurate threshold value of *E*_GSH_ (approximately −285 mV) needed to activate HIV-1 in U1 cells. Moreover, co-treatment of U1 cells with BSO and H_2_O_2_ synergistically reactivates HIV-1, a finding consistent with an earlier report of effective reactivation of HIV-1 using a combination of nontoxic concentrations of histone deacetylase inhibitors and BSO (61). Although this has to be verified in multiple models of HIV-1 latency, we believe that this bioprobe can solve a major technological hurdle in setting up high throughput screens for redox-based compounds to precisely manipulate *E*_GSH_ for reactivation and elimination of HIV-1 in combination with HAART without triggering global ROS-mediated toxicity.

Numerous studies have indicated that viruses induce oxidative stress as a strategy to promote their own expression and replication through redox-mediated chromatin remodeling and activation of transcription factors such as NF-κB (1, 62, 63). We verified these results and confirmed that activation of HIV-1 replication by diverse agents uniformly induces an oxidative shift in *E*_GSH_ (−240 mV). Furthermore, we corroborated earlier studies demonstrating opposing effects of GSH inhibitor (BSO) or precursor (NAC) on HIV-1 reactivation (61, 64). These findings are important because of conflicting reports showing variable levels of GSH in cells and tissues of HIV-infected people (65–67). Similarly, some studies found that NAC was ineffective in restoring GSH levels in lymphocytes and plasma of AIDS patients (68). Finally, it is difficult to reconcile why patients on HAART show heightened oxidative stress despite considerably reduced viral load (69, 70). In this context, we believe that Grx1-roGFP2 provides a more reliable tool to image redox signaling and opens up new avenues of research to understand the mechanisms of antioxidant and drug action during HIV-1 infection.

To investigate whether Grx1-roGFP2 can be used to monitor changes in *E*_GSH_ of macrophages infected with other human pathogens, we infected U937 cells with laboratory-adapted and clinical isolates of *M. tuberculosis*. Interestingly, infection with *M. tuberculosis* strain generated considerable oxidative shift in *E*_GSH_ of macrophages., which agrees with studies demonstrating *M. tuberculosis-*induced depletion of intramacrophage GSH during infection (71). Another new finding is the potential of surface-exposed and secretory immunomodulatory polyketide lipids produced by *M. tuberculosis* strains in inducing oxidative *E*_GSH_ in macrophages. As expected, *M. tuberculosis* lipids also increased virus activation via modulating *E*_GSH_. Importantly, lipids from the MYC 431 XDR strain were powerful inducers of oxidative stress and HIV-1 reactivation. Consistent with our finding, a recent report has shown the importance of a strain-specific polyketide lipid (phenolic glycolipid-1 (PGL-1)) produced by *M. tuberculosis* in influencing cytokine production and NF-κB activation to modulate HIV-1 replication (72). Our data indicate that clinical drug-resistant *M. tuberculosis* strains may harbor important genotypic and phenotypic variations to generate a permissive host redox environment for catalyzing infection, replication, and co-habitation by these two intracellular human pathogens.

**FIGURE 13.**
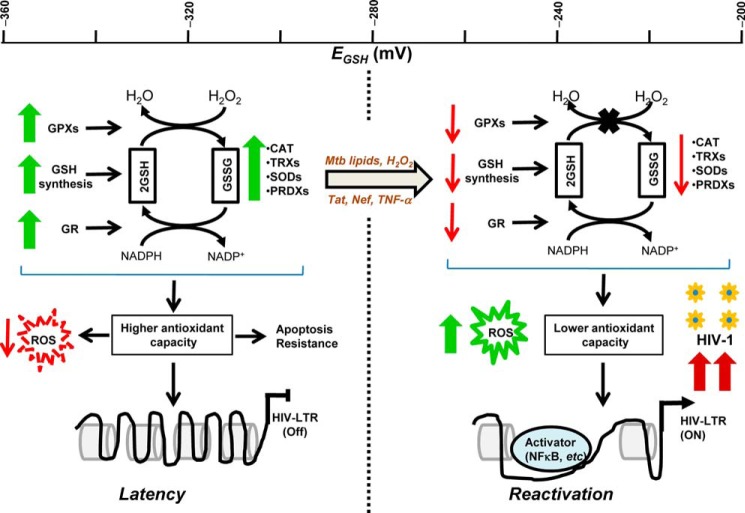
**Oxidoreductive stress model of HIV-1 persistence and reactivation.** The model suggests that increased expression of genes involved in glutathione biosynthesis, GSH-assisted H_2_O_2_ detoxification (*GPXs*), GSSG reduction (*GR*), and other cellular antioxidants together contribute toward maintenance of reductive subcellular *E*_GSH_ (approximately −320 mV) and enhanced capacity to resist oxidative stress and apoptosis in the cells latently infected with HIV-1. A moderate oxidative shift in *E*_GSH_ (−285 mV) induced by H_2_O_2_ or *M. tuberculosis* lipids reactivates HIV-1 from persistence. Active HIV-1 replication stimulated by viral proteins (Tat and Nef) or cytokines (TNF-α) generates excessive oxidative shift in *E*_GSH_ (more than −240 mV), thereby promoting a further increase in transcription from HIV-1 LTR through redox-dependent transcription factors such as NFκB, and eventual progression to AIDS. The *scale bar* at the top denotes *E*_GSH_ values. The *thick green colored arrows* and *thin red colored arrows* denote higher and lower expression, respectively. *CAT*, catalase.

We show that the *E*_GSH_ response varies during latent and reactivation phases of infection. Interestingly, our array analysis revealed that altered expression of genes involved in redox homeostasis and signaling could be the source of this variability. Induction of antioxidant genes during virus latency indicates that U1 cells have gained oxidative stress scavenging capabilities, which is in agreement with our findings showing greater oxidative stress response and anti-apoptotic behavior of these cells. These changes in redox physiology could result either from direct action of HIV-1 gene products expressed or repressed in latency or were selected during the process of isolation of a chronically infected U1 clone from the infected U937 cells. Along these lines, it is important to note that one of the main HIV proteins, Tat, which is known to induce oxidative stress by interfering with the expression of host antioxidant genes (73–75), is rendered nonfunctional in U1 due to mutations (76). These observations along with our results showing Tat-mediated activation of HIV-1 and oxidative shift in *E*_GSH_ point toward a redox-modulatory role of Tat in regulating HIV-1 latency and reactivation. Regardless of the exact mechanisms, the differences in *E*_GSH_ and redox gene expression may play a role in the maintenance of HIV latency. In line with this argument, many of the redox genes up-regulated in U1 cells are known HIV-1-suppressive factors. For example, a higher expression of genes encoding the chemokine CCL5, heme oxygenase-1 (HMOX-1), thioredoxin reductase, peroxiredoxins, glyceraldehyde-3-phosphate dehydrogenase (GAPDH), etc. are host defense mechanisms well known to negatively regulate HIV-1 infection and replication (77–81). Based on this, it is reasonable to hypothesize that the products of some of the redox genes may be involved in maintaining HIV-1 latency. Therefore, treating latently infected cells with agents targeting specific redox genes/pathways may be considered as an alternative approach to eject the virus from latent reservoirs. In agreement with this notion, most of the redox genes that were up-regulated during latency showed dramatic down-regulation during the active phase of replication. In general, expression data suggest that pro-oxidative conditions are permissive for viral replication, whereas greater oxidative stress buffering capacity supports latency. Based on these findings, we provide a redox-based model of HIV-1 latency and activation (Fig. 13). Although our study was restricted to U1 and U937 cells, variations in the expression of a small number of redox genes during latency and reactivation show similar changes in other chronically infected cell lines such as ACH-2 and J1.1 (82). Similarly, proteomics of monocytes/macrophages derived from HIV-1-infected patients showed depressed antioxidants such as thioredoxins, peroxiredoxin, myeloperoxidase, superoxide dismutase, ferritin heavy chain, etc. (83, 84). Finally, a direct biological relevance of our study emerges from expression data showing repressed expression of key antioxidant genes in the PBMCs of symptomatic HIV patients.

In summary, we demonstrate that Grx1-roGFP2 is a sensitive, specific, and reliable bio-tool to quantify compartment-specific redox changes associated with HIV-1-infected and HIV- *M. tuberculosis* co-infected cells. Our study indicates that this biosensor will be extremely useful in investigating physiological changes associated with HIV-1 infection, mechanisms of drug action and drug resistance, and screening small molecule modulators of redox potential to expose HIV-1 from latency. Because redox stress and signaling is central to several chronic viral and bacterial infections, accurate mathematical indicators of the redox state, as revealed in this study, may provide specific information on how much redox machinery can be manipulated to achieve desirable therapeutic effects.

## Supplementary Material

Supplemental Data

## Footnotes

3 The abbreviations used are:

ROS: reactive oxygen species
RNS: reactive nitrogen species
roGFP: redox-sensitive green fluorescent protein
PBMC: peripheral blood mononuclear cells
PMA: phorbol 12-myristate 13-acetate
BSO: l-buthionine sulfoximine
PI: propidium iodide
NAC: *N*-acetylcysteine
NEM: *N*-ethylmaleimide
PFA: paraformaldehyde
HAART: highly active antiretroviral therapy
GPX: glutathione peroxidase
DCF-DA: dichlorofluorescein diacetate
DCF: dichlorofluorescein
DCFH: 2′,7′-dichlorodihydrofluorescein
qRT: quantitative RT-PCR.
