# Supplementary material for: Measuring Glutathione Redox Potential of HIV-1-infected Macrophages
Source: J Biol Chem. 2014 Nov 18;290(2):1020–38. doi: 10.1074/jbc.M114.588913 (PMC4294471; doi:10.1074/jbc.M114.588913)
Supplement: Supplemental Data [file supp_290_2_1020__index.html]

Measuring Glutathione Redox Potential of HIV-1 Infected Macrophages — Measuring Glutathione Redox Potential of HIV-1-infected Macrophages — Redox Potential of HIV-1-infected Macrophages — Supplemental Data 

# Measuring Glutathione Redox Potential of HIV-1-infected Macrophages

## Supplemental Data

**Files in this Data Supplement:**

- Table S1 (.xlsx, 44 KB) - Expression profile of oxidative stress responsive genes in U1 and U937 under untreated and PMA treated conditions
